# Resistance of *Klebsiella pneumoniae* Strains Carrying *bla*_NDM–1_ Gene and the Genetic Environment of *bla*_NDM–1_

**DOI:** 10.3389/fmicb.2020.00700

**Published:** 2020-04-30

**Authors:** Tianxin Xiang, Chuanhui Chen, Jiangxiong Wen, Yang Liu, Qi Zhang, Na Cheng, Xiaoping Wu, Wei Zhang

**Affiliations:** ^1^Department of Hospital Infection Control, The First Affiliated Hospital of Nanchang University, Nanchang, China; ^2^Department of Respiratory and Critical Care, The First Affiliated Hospital of Nanchang University, Nanchang, China; ^3^Department of Infectious Disease, The First Affiliated Hospital of Nanchang University, Nanchang, China

**Keywords:** carbapenem resistance, NDM-1, *Klebsiella pneumoniae*, plasmid, genetic characteristics

## Abstract

**Objective:**

Regional dissemination is the major cause of the widespread prevalence of a plasmid-encoding NDM-1 enzyme. We investigated the drug resistance, joint efficiency, and gene environment of a *Klebsiella pneumoniae* strain carrying *bla*_NDM–1_ gene.

**Materials and Methods:**

Carbapenem-non-susceptible strains were analyzed using the VITEK 2 Compact. Strains carrying *bla*_NDM–1_ were identified using polymerase chain reaction and sequencing. Antimicrobial susceptibility testing and plasmid conjugation experiments were then conducted. Strains carrying *bla*_NDM–1_ were subjected to Southern blot analysis. After the gene mapping of *bla*_NDM–1_, library construction, and sequencing, plasmids were subsequently spliced and genotyped using the software Glimmer 3.0, and then analyzed using Mauve software.

**Results:**

Among 1735 carbapenem-non-susceptible strains, 54 strains of *bla*_NDM–1_-positive bacteria were identified, which consisted of 44 strains of *K. pneumoniae*, 8 strains of *Acinetobacter baumannii* and 2 strains of *Escherichia coli*. Strains carrying *bla*_NDM–1_ had a resistance rate of more than 50% in most antibiotics. Plasmid conjugation between strains carrying *bla*_NDM–1_ and *E. coli* strain J53 had a success rate of 50%. Southern blot analysis indicated that each strain had multiple plasmids containing *bla*_NDM–1_. Among the five plasmids containing *bla*_NDM–1_ in *K. pneumoniae* for sequencing, two plasmids with complete sequences were obtained. The findings were as follows: (i) The p11106 and p12 plasmids were highly similar to pNDM-BTR; (ii) the p11106 and p12 plasmids showed differences in the 20–30 kb region (orf00032–orf00043) from the other six plasmids; and (iii) *bla*_NDM–1_ was located at orf00037, while *ble* was found at orf00038. Two *tnpA* genes were located in the upstream region, and orf00052 (*tnpA*) in the 36 kb region was in the downstream sequence.

**Conclusion:**

*bla*_NDM–1_-containing bacteria exhibit multidrug resistance, which rapidly spreads and is transferred through efficient plasmid conjugation; the multidrug resistance of these bacteria may be determined by analyzing their drug-resistant plasmids. The presence of *ble* and *tnpA* genes suggests a possible hypothesis that *bla*_NDM–1_ originates from *A. baumannii*, which is retained in *K. pneumoniae* over a long period by transposition of mobile elements.

## Introduction

The clinical application of sulfa drugs can be traced back to the 1930s, which marked a new era of antimicrobial therapy. Once exposed to antibacterial drugs, bacteria spontaneously change their metabolic pathways or produce corresponding inactivating substances to resist antibiotics, exhibiting drug resistance. Notably, the abuse of antibiotics poses selective pressure of bacteria, conferring a survival advantage on drug-resistant bacteria. Consequently, numerous drug-resistant bacteria are spread in different pathogens.

New Delhi metallo-β-lactamase1 (NDM-1), also known as metallo-β-lactamase or metal-β-lactamase, was first isolated from a highly infectious and pathogenic multidrug-resistant strain of *Klebsiella pneumoniae* in 2009 (Yong et al., 2009). Cases of infection with the *bla*_NDM–1_ gene have subsequently been reported in more than 20 countries and regions worldwide, including the United Kingdom and India (Kumarasamy et al., 2010). The therapeutic efficacy of multiple antibiotic treatments for bacteria carrying *bla*_NDM–1_ is usually unsatisfactory. Therefore, bacterial strains carrying *bla*_NDM–1_ are also called superbugs. With a prolonged length of stay, bacteria-carrying *bla*_NDM–1_ have a higher probability of being isolated from stool samples. However, infection with *bla*_NDM–1_ cannot be determined from clinical symptoms and signs (Bush and Fisher, 2011). Superbugs pose a serious challenge to antibiotic therapy.

The *bla*_NDM–1_ gene is mainly distributed in plasmids and occasionally in the chromosomes of *Escherichia coli*, *Pseudomonas aeruginosa*, and *Proteus mirabilis* (Girlich et al., 2015; Rahman et al., 2015; Shen et al., 2017). In clinical practice, *bla*_NDM–1_ plasmids from different bacterial species isolated from the same patient typically have a similar structure, suggesting the significance of plasmids in the spread of *bla*_NDM–1_. Plasmids containing *bla*_NDM–1_ vary in size from 30–300 kb, and exist in different types, such as IncA/C, IncL/M, and IncR (Carattoli et al., 2015; Gamal et al., 2016). Specifically, IncA/C has a wide range of hosts and can exist in multiple strains, such as *Enterobacteriaceae*, *Pseudomonas*, *Acinetobacter*, and *Vibrio cholerae*, providing convenience for *bla*_NDM–1_ in different species of bacterial hosts (Wailan and Paterson, 2014).

The sequences and genetic modes of *bla*_NDM–1_ have been identified in previous studies. However, the *bla*_NDM–1_ gene environment is yet to be determined. In addition, studies mostly focus on *bla*_NDM–1_ in *Acinetobacter* (Bontron et al., 2016; Wang et al., 2017) and rarely on *bla*_NDM–1_ carried by *K. pneumoniae*. In the present study, 1735 carbapenem-non-susceptible bacteria were collected from The First Affiliated Hospital of Nanchang University. These strains were sequenced, conjugated, and compared with the corresponding plasmids. This study aimed to analyze the differences in the *bla*_NDM–1_ gene environment and provide directions in clarifying the origin and propagation of the *bla*_NDM–1_ gene.

## Materials and Methods

### Sample Collection and Identification

Approval was obtained from the Medical Ethics Committee of The First Affiliated Hospital of Nanchang University and informed consent was obtained from each subject. Carbapenem-non-susceptible bacteria were then collected from The First Affiliated Hospital of Nanchang University from January 2013 to December 2016. A total of 1735 carbapenem-non-susceptible bacteria were isolated and then identified using VITEK 2 Compact (Pioneering Diagnostics, France) at the Microbiology Laboratory at The First Affiliated Hospital of Nanchang University.

### Polymerase Chain Reaction and Sequencing

Polymerase chain reaction (PCR) templates were prepared by boiling. Fresh bacteria were harvested and diluted in 500 μL ddH_2_O for 10 min in a boiling water bath. The supernatant was collected as PCR templates. Subsequently, a PCR system with a total volume of 50 μL was prepared, consisting of 25 μL of Taq Mix (Takara, Dalian), 2 μL of forward primer, 2 μL of reverse primer, 2 μL of template, and 19 μL of ddH_2_O. Subsequently, 29 cycles of PCR were conducted. The *bla*_NDM–1_ primer sequences were forward 5’-GGCGGAAGGCTCATCACGA-3’ and reverse 5’-CGCAACACAGCCTGACTTTC-3’. The amplified product was 287 bp.

The PCR products were analyzed using electrophoresis with 1% agarose gel and 1 × TAE at 120 V for 25 min. Strains verified by sequencing to contain *bla*_NDM–1_ were used as markers. Electrophoresis results were obtained using an ultraviolet (UV) transilluminator. Positive PCR products were sequenced (Synbio Technology, Suzhou, China), and the sequencing results were compared using the software BLAST. Strains that were positive for PCR and matched the sequencing results were identified as those containing *bla*_NDM–1_.

### Antimicrobial Susceptibility Testing

Antimicrobial susceptibility testing was conducted using the zone of inhibition test, and the comprehensive drug resistance of each antibiotic was determined using the *E*-test. The antibiotics screened in this study included imipenem, meropenem, ertapenem, amikacin, amoxicillin/clavulanicacid, aztreonam, ceftriaxone, ceftazidime, cephalosporins, cefoxitin, cefazolin, ciprofloxacin, gentamicin, levofloxacin, piperacillin/tazobactam, trimethoprim/sulfamethoxazole, tetracycline, ticarcillin/clavulanicacid, and tobramycin.

### Plasmid Conjugation

The receptor strain was sodium azide-resistant *E. coli strain* J53. The donor and receptor strains were implanted in the Mueller-Hinton plate and cultured at 37°C for 16–18 h. Strains in appropriate amounts were inoculated in a glass tube containing 5 mL of LB medium and then cultured at 37°C for 16–18 h. Subsequently, 400 μL of the donor strain and 200 μL of the receptor strain were added to a glass tube containing 800 μL of LB broth medium and then cultured at 37°C for 16–18 h. Meanwhile, the donor strain, screened in 180 μg/mL sodium azide, and the receptor strain, screened in 0.5 μg/mL of imipenem, were used as blank controls. Exactly 100 μL of the aforementioned mixture was added to the Mueller-Hinton plate and cultured at 37°C for 16–18 h. Conjugation strains in good condition were ultimately identified using the VITEK 2 compact automatic microbial identification instrument.

### Southern Blot Analysis

Southern blot analysis was conducted using 1% agarose gel with 1 × TAE and run on 120 V electrophoresis for 40 min. The gel was incubated in 0.25 mol/L HCl for 15 min, 0.5 mol/L NaOH for 20 min twice, and 0.1 mol/L phosphate buffer for 15 min twice. Membrane transfer was conducted in a 20 × saline-sodium citrate buffer. The membrane was washed in a 2 × saline–sodium citrate buffer and then dried in a baking oven at 80°C for 2 h. PCR products carrying *bla*_NDM–1_ were labeled using the DIG High Prime DNA Labeling and Detection Starter Kit I (Roche, United States). DIG-labeled DNA products were prepared and examined using Southern blot analysis, and images were obtained using a UV transilluminator.

### Plasmid Sequencing

Five qualified plasmids containing *bla*_NDM–1_ were used to construct a sequencing library. These 5 vectors were from 4 strains, which showed high resistance to the antibiotics and successfully conjugated with *E. coli* J53. The data are provided as Supplementary Material and Supplementary Table S2. Briefly, 1 μg of plasmid was placed in a Covaris tube, and the DNA was separated into 400 bp fragments using Covaris S2 (Covaris, United States). Small DNA fragments were generated for library construction using the NEXTflex DNA Sequencing Kit compatible with Biomek FXp (Bio Scientific, United States). The library fragments were subjected to paired-end sequencing (2 × 150 bp) on the HiSeq2500 Sequencing System (Illumina, United States).

Clean reads after pre-processing were assembled using Velvetver.1.2.03 software. Gene prediction and annotation analyses were performed using Glimmer 3.0 software. The p11106 and p12 plasmids were compared with the plasmid sequences without *bla*_NDM–1_ of the seven species. Similarities in the plasmid sequences were depicted using Mauve software. Gene functions in different regions were annotated and analyzed.

## Results

### Screening and Identification of Strains Carrying *bla*_NDM–1_

Among the 1735 carbapenem-non-susceptible strains harvested in this experiment, 54 strains (3.1%) were *bla*_NDM–1_-positive. These strains consisted of 44 strains of *K. pneumoniae*, 8 strains of *A. baumannii*, and 2 strains of *E. coli*. The *bla*_NDM–1_ gene was not found in *P. aeruginosa*, *Enterobacter cloacae*, *Bacillus*, *Maltophilia*, or *Pseudomonas cepacia.* All sequencing results of the 54 strains were consistent with the NCBI database^1^. The positive rates of each strain are listed in Table 1.

**TABLE 1:** Positive rate of the *bla*_NDM–1_ gene in each strain.

**Strain name**	**Sample size**	**Positive number**	**Positive rate**
*Klebsiella pneumoniae*	935	44	4.7%
*Acinetobacter baumannii*	546	8	1.5%
*Escherichia coli*	92	2	2.1%
*Pseudomonas aeruginosa*	83	0	0
*Bacillus*	40	0	0
*Maltophilia*	20	0	0
*Pseudomonas cepacia*	19	0	0
Total	1735	54	3.1%

These 54 multidrug-resistant strains were obtained from 43 patients at The First Affiliated Hospital of Nanchang University from January 2013 to December 2016. The patients were from different cities and provinces. Temporal and regional differences suggested that the same strains could not be causing the outbreak. Patient records are shown in Supplementary Table S1.

### Determination of Drug Resistance

**FIGURE 1:**
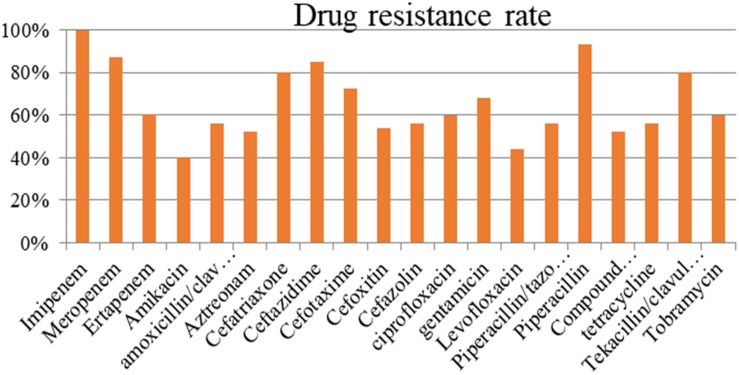
Drug resistance in *bla*_*NDM–1*_-positive strains.

The resistance rate of the strains carrying *bla*_NDM–1_ exceeded 50% in most antibiotics (Figure 1). The tested antibiotics exhibited nearly 100% resistance to imipenem and more than 90% resistance to meropenem and piperacillin. Meanwhile, the *bla*_NDM–1_-positive strains showed the lowest resistance (40%) to amikacin.

### Plasmid Conjugation

The plasmid conjugation experiment was conducted on all *bla*_NDM–1_-positive strains and J53. A total of 27 strains (21, *K. pneumoniae* strains; 5, *A. baumannii* strains; and 1, *E. coli* strain) successfully conjugated their plasmids containing the *bla*_NDM–1_ gene (Table 2). The success rate of plasmid conjugation was 50%.

**TABLE 2:** Plasmid conjugation of strains.

**Strain**	**Number of**	**Number**	**Rate of successful**
**name**	***bla*_NDM–1_ strains**	**conjugated**	**conjugation**
*Klebsiella pneumoniae*	44	21	47.7%
*Acinetobacter baumannii*	8	5	62.5%
*Escherichia coli*	2	1	50.0%
Total	54	27	50.0%

### Location of the *bla*_NDM–1_ Gene

**FIGURE 2:**
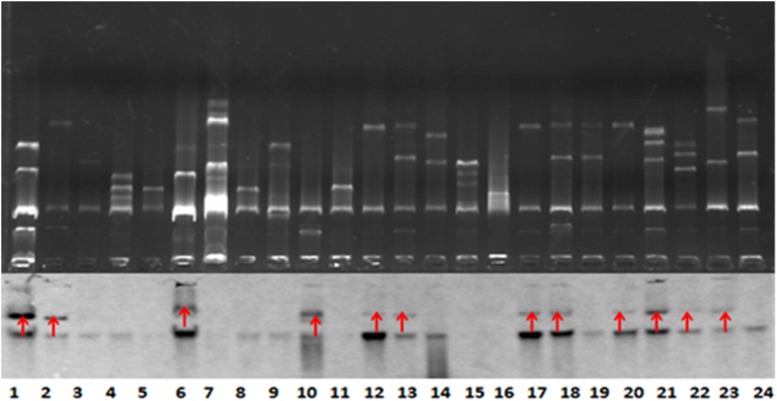
Southern blot analysis for *bla*_*NDM–1*_ localization. The conjugation of *bla*_*NDM–1*_-positive strain J53 was visualized. The 12 strains are denoted by red arrows. Lanes 12, 13, 17, 18, 20, 21, 22, and 23 are visualized bands that indicate *Klebsiella pneumoniae* strains carrying *bla*_*NDM–1*_. Lanes 6 and 10 indicate *Acinetobacter baumannii* strains carrying *bla*_*NDM–1*_. Lanes 1 and 2 indicate *Escherichia coli* strains carrying *bla*_*NDM–1*_. The lower part of this figure is mirrored with the top part.

*Bla*_NDM–1_-positive strains that were successfully conjugated were subjected to Southern blot analysis to detect the location of *bla*_NDM–1_. The results further demonstrated the conjugation of *bla*_NDM–1_ in *E. coli* strain J53 (Figure 2).

### Sequencing of Plasmids

Plasmid libraries were constructed from genomic DNA. Qualified plasmid DNAs were sequenced, and the results suggested excellent qualifications for library construction and sequencing (Table 3). After removal of DNA from host genes and other plasmid DNAs, approximately 20% of the reads were extracted from target plasmid DNAs with over 2000× coverage.

**TABLE 3:** Quality and coverage statistics of plasmid genome sequencing.

**Plasmid**	**Raw reads (pair)**	**Clean reads (pair)**	**Q20 (%)**	**Q30 (%)**	**Target plasmid reads (pair)**	**Coverage**
p11106	5,581,280	5,567,524	95.94	91.06	1,523,524 (27.3%)	7,109
p12	4,562,335	4,551,783	95.05	89.62	1,157,414 (25.4%)	5,401
p243323	4,707,263	4,695,911	96.24	91.56	967,971 (20.6%)	4,517
p32	7,672,469	7,653,464	96.09	91.27	1542,915 (20.1%)	7,200
p7-1973	3,549,640	3,541,667	93.99	87.93	909,991 (25.6%)	2,316

Subsequently, the clean reads of each plasmid were synthesized using Velvet. Large-fragment sequence assembly is presented in Table 4. Although coverage was sufficiently high (>2000×), the synthesis was not satisfactory, with the contigs between 33 and 161. Thus, artificial gap closing with the KU862632.1 strain was conducted using the Cytoscape platform, and two complete plasmid (p12 and p11106) sequences were generated. Synthesis of p243323, p32, and p7-1973 failed (Table 4).

**TABLE 4:** Statistics of plasmid synthesis.

**Plasmid**	**Initial contigs**	**N50**	**Synthesis**	**Completed**	**GC (%)**
p11106	68	4,477	9	Yes	51.99
p12	161	2,403	16	Yes	51.99
p243323	42	7,920	19	No	51.72
p32	33	15,996	16	No	51.74
p7-1973	146	2,914	25	No	53.23

**FIGURE 3:**
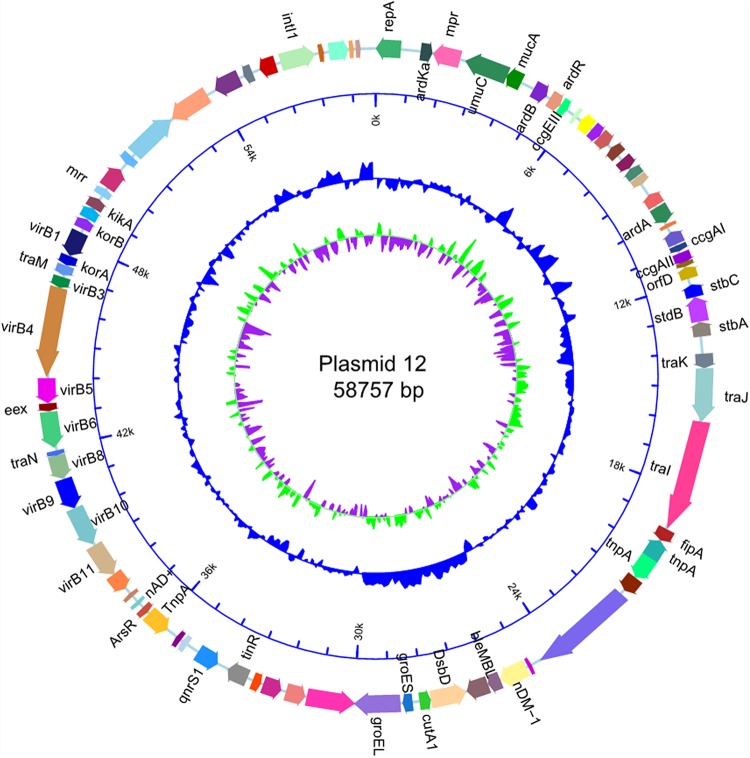
Map of plasmid p12 sequence.

The size of p12 was the same as that of p11106 (58757 bp). Cytosine was located on 50396 bp of p11106, thymine was located in p12, and the remaining regions were the same. In the following gene prediction and annotation analysis, p12 was used as an example. As depicted in Figure 3, 46 genes were reverse transcribed, and 39 were forward transcribed without chain specificity (*p* = 0.627). We uploaded the sequencing data to the NCBI database under the SRA (Sequence Read Archive) accession number PRJNA596354.

### Comparative Analysis of the bla_NDM–1_ Gene Environment

**FIGURE 4:**
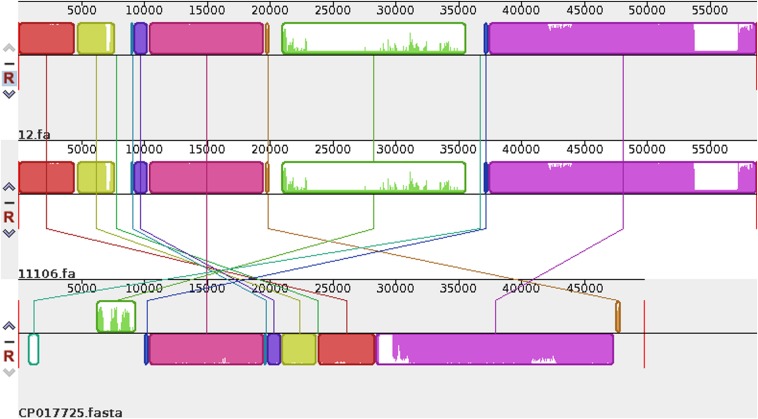
Comparison between p12 and p11106 with pSARB26_02. Differences between p12 and p11106 with pSARB26_02 (CP017725) were mainly enriched in the 20–37 kb region (green region), with orf00032–orf00052 in p11106 and p12. Two *TnpA* transposases are located in this region (orf00032 mapped 20169–19597 bp, negative strand; orf00033 mapped 20160–20864 bp, positive strand), indicating that gene insertion is highly possible in this region of p11106 and p12. Similarly, additional 3.5 kb sequences present in the 3’ UTR of p11106 and p12 were mapped orf00078–orf00082. This may also be explained by gene transposition or insertion.

Plasmid sequences from seven species were subjected to similarity analysis using Mauve software. The baseline characteristics of the selected plasmids are listed in Table 5. p11106 and p12 were compared with the aforementioned plasmids, and their similarity information is depicted in Figure 4 and Table 6.

**TABLE 5:** Plasmid information.

**ID**	**Species**
KF534788.1	*Escherichia coli* plasmid pNDM-BTR, complete sequence
CP017725.1	*Salmonella enterica* subsp. enterica serovar Stanleyville str. CFSAN000624 strain SARB61 plasmid pSARB26_02, complete sequence
HM126016.1	*Klebsiella oxytoca* plasmid pKOX105, complete sequence
KM660724.1	*Morganella morganii* strain MRSN22709 plasmid pMR3-OXA181, complete sequence
KT989598.1	*Enterobacter cloacae* strain CRE1506 plasmid pIMP-SH1506, complete sequence
KU051710.1	*Citrobacter freundii* strain CRE1503 plasmid pIMP-FJ1503, complete sequence
KU862632.1	*Klebsiella pneumoniae* strain CRE1495 plasmid pIMP-KP1495, complete sequence

**TABLE 6:** Comparison of differential plasmids.

**Plasmid**	**Inserted region (Gene)**	**Deleted region (Gene)**
pNDM-BTR	38 kb (*TnpA*)	
pSARB26_02	/	20–37 kb (orf00032–orf00052) 53–57.5 kb (orf00078–orf00082)
pKOX105	/	7–10 kb (orf00012–orf00017) 21–29 kb (orf00035–orf00042)
pMR3-OXA181	24.5–30 kb (orf00036–orf00043)	/
pIMP-SH1506	27–30 kb, 48–51 kb, and 52–53 kb	19.5–30 kb (orf00032–orf00043)
pIMP-FJ1503	45–47.5 kb,48.5–49 kb	19.5–30 kb (orf00032–orf00043)
pIMP-KP1495	45–48 kb	21–30 kb (orf00034–orf00043)

In addition to the plasmid pNDM-BTR, differences between p11106 and p12, and the remaining seven plasmids were mainly enriched in the 20–30 kb region. Although the plasmid pMR3-OXA181 was not absent in this region, it exhibited a considerably low homology to p11106 and p12. The corresponding genes mapping this 10 kb region were orf00032–orf00043. Moreover, gene annotation showed that two genes (*TnpA* and a mobile element protein) with similar functions were located in the upstream sequences of this region. *Bla*_NDM–1_ was also located in these sequences (orf00037). *IMP-4* and β*-lactamase* were absent in this region. The gene annotations of orf00032–orf00043 are presented in Table 7.

**TABLE 7:** Gene annotation of orf00032–orf00042.

**Gene**	**Start**	**End**	**Strand**	**Protein length**	**Function**
orf00032	20169	19597	–	190	*tnpA*
orf00033	20160	20864	+	234	*tnpA*
orf00034	20870	21352	+	160	Mobile element protein
orf00035	21485	24502	+	1005	Mobile element protein
orf00036	24873	24757	–	38	Multispecies: hypothetical protein
orf00037	24931	25743	+	270	NDM-1
orf00038	25747	26112	+	121	*ble*_MBL_
orf00039	26117	26755	+	212	Phosphoribosylanthranilate isomerase
orf00040	27797	26766	–	343	DsbD
orf00041	28131	27802	–	109	*cut*_A1_
orf00042	28325	28615	+	96	*gro*_ES_

As shown in Table 7, (i) the p11106 and p12 plasmids were highly similar to pNDM-BTR; (ii) the p11106 and p12 plasmids showed differences in the 20–30 kb region (orf00032–orf00043) from the other six plasmids; and (iii) *bla*_NDM–1_ was located in the middle of the orf00032–orf00043 region, whereas *ble* was found nearby. Two *tnpA* genes were on the top, and orf00052 (*tnpA*) in the 36 kb region was in the downstream sequence.

## Discussion

Among the 1735 carbapenem-non-susceptible strains, 54 (3.1%) of *bla*_NDM–1_-positive bacteria were identified, consisting of 44 strains (4.7%) of *K. pneumoniae*, 8 strains (1.5%) of *A. baumannii*, and 2 strains (2.1%) of *E. coli*. Our findings are consistent with previous studies showing that *bla*_NDM–1_ is mainly carried by *K. pneumoniae*, *E. coli, A. baumannii*, and *E. cloacae* (Deshpande et al., 2010; Rolain et al., 2010; Zarfel et al., 2011; Dongmei et al., 2017). Among the collected strains carrying *bla*_NDM–1_ in our hospital, *K. pneumoniae* (4.7%) had the highest positive rate, which was inconsistent with other reports that identified *Acinetobacter* and *Klebsiella as* the major *bla*_NDM–1_-positive bacteria (Chen et al., 2014; Zhang et al., 2014; Shengshu et al., 2015). Both *Acinetobacter* and *Klebsiella* are significant in the regional dissemination of *bla*_NDM–1_-positive bacteria. *K. pneumoniae* could be the vital preservation host of *bla*_NDM–1_.

Antimicrobial susceptibility testing indicated that NDM-1-resistant bacteria exhibited strong resistance to most antibiotics (except for amikacin), with a total resistance rate above 50%. The drug resistance of NDM-1 to aminoglycosides, β-lactam antibiotics/β-lactamase inhibitors, and carbapenems are relatively high (Shengshu et al., 2015). NDM-1 has a drug resistance of up to 100% for dolipenem, ampicillin, furadantin, cefazolin, cefuroxime, cefotaxime, ceftriaxone sodium, ceftazidime, and cefoxitin. Its drug resistance rates to some carbapenems are as follows: meropenem, 97.30%; imipenem, 97.30%; and ertapenem, 92.00%. However, the drug resistance rates of NDM-1 to polymyxin B (37.93%), amikacin (32.65%), tigecycline (7.69%), and polymyxin E (2.33%) are significantly lower, compared with other antibiotics. NDM-1-resistant bacteria might be mostly multidrug-resistant, and plasmids containing *bla*_NDM–1_ can perform multiple drug-resistant gene transfers (Barguigua et al., 2015; Sarkar et al., 2015; Villa et al., 2015; Liu et al., 2016).

Our experiment obtained 27 successfully conjugated J53 strains resistant to sodium azide, which is consistent with previous studies (Wailan et al., 2015; Kocsis et al., 2016). Conjugation with *K. pneumoniae*, *A. baumannii*, *and E. coli* as donor plasmids containing *bla*_NDM–1_ achieved a success rate of approximately 50%. Southern blot analysis confirmed that *bla*_NDM–1_ was mainly expressed in plasmids, and each drug-resistant strain carrying *bla*_NDM–1_ could contain multiple *bla*_NDM–1_-positive plasmids. The *bla*_NDM–1_-positive plasmids exhibited a relatively strong ability for conjugation transfer. Specifically, IncA/C showed adaptation to a wide range of hosts. It can be found in *Enterobacteriaceae*, *Pseudomonas*, *Aeromonas*, *Vibrio cholera*, and other bacterial genera, and can be transmitted within or between strains through conjugation (Wailan and Paterson, 2014; Carattoli et al., 2015; Gamal et al., 2016).

Sequencing of five plasmids obtained from the collected *K. pneumoniae strains* was highly qualified (coverage >2000×). Regardless, the synthesis of p243323, p32, and p7-1973 failed; only p11106 and p12 were successfully synthesized. This result could be explained by the complexity of the plasmid structure and interruption of the host genome and other plasmids during plasmid extraction. Among the sequencing reads, only 20–30% of the plasmids were identified to carry the*bla*_NDM–1_ gene, significantly increasing the difficulty of synthesis. The only difference between p11106 and p12 was the 50396 bp (orf00074 was mapped) region, where c.587T > C occurred in p11106 and thus resulted in p.196Ile > Thr. A total of 85 genes were contained in the p11106 sequence, and most of them had functions that could be identified using BLAST.

The structures of plasmids p11106 and p12 were similar, and their strains *Kp*.11 and *Kp*.12 were also similar in resistance (Supplementary Table S2). This finding suggests that resistant plasmids are a key factor in determining the drug resistance of strains.

The genetic environment of a certain gene helps reveal the origin and genetic characteristics of the gene. Poirel et al. (2011) uncovered the full-length or truncated IS*Aba125* sequences in the upstream region of *bla*_NDM–1_ and conserved bleomycin (*ble*) resistance genes in the downstream region. They hypothesized that IS*Aba125* sequences are carried by *bla*_NDM–1_ when it transfers from the original host, and the *bla*_NDM–1_ and *ble* genes come from the same original host strain (Poirel et al., 2011). In 2012, Toleman analyzed all available *bla*_NDM–1_-associated sequences and found that IS*Aba125* sequences are always present in the 100 bp upstream region of *bla*_NDM–1_, and demonstrated that *bla*_NDM–1_ is a chimera. The chimeric process occurs in *A. baumannii* and is mediated by IS*Aba125* (Jones et al., 2014, 2015; Toleman et al., 2015). Our sequencing results identified certain differences in the 20–30 kb region between p11106, p12, and the compared plasmids, except for pNDM-BTR. On the basis of the aforementioned findings, we propose the following: (a) p11106 and p12 are structurally similar to pNDM-BTR, indicating that p11106 and p12 may present characteristics of pNDM-BTR (McGann et al., 2015); (b) the differences in the 20–30 kb region between p11106, p12, and the compared plasmids support the theory of gene polymorphism (Potron et al., 2011; Mata et al., 2012; Datta et al., 2017); (c) the existence of *ble* indicates that *bla*_NDM–1_ may originate from *A. baumannii*; and (d) the presence of two *TnpA* in the upstream region of *bla*_NDM–1_ and orf00052 (*TnpA*) in the 36 kb downstream region suggests that *bla*_NDM–1_ is acquired from plasmid transposition and long-term preservation (Campos et al., 2015; An et al., 2016; Khong et al., 2016).

This experiment screened drug-resistant strains carrying *bla*_NDM–1_ gene from carbapenem-non-susceptible bacteria collected from The First Affiliated Hospital of Nanchang University between January 2013 and December 2016. We assessed their drug resistance and plasmid conjugation transfer ability. Two complete plasmid sequences were obtained after sequencing analysis of five *bla*_NDM–1_-positive plasmids isolated from *K. pneumoniae* and compared with *bla*_NDM–1_-negative plasmids with known sequences. However, data on abundance were limited owing to the small sample size of drug-resistant strains as well as complexity and interferences during plasmid synthesis. In addition, the conclusion drawn from sequencing in this study is yet to be verified by molecular experiments. Gene deletion (*IMP-4* and β*-lactamase*) occurred in p11106 and p12. The presence of a potential relationship between gene deletion and *bla*_NDM–1_ requires further study.

## Conclusion

In conclusion, NDM-1-resistant bacteria exhibit multidrug resistance and spread to a certain extent in the hospital via efficient plasmid conjugation transfer. *K. pneumoniae* may be an important intermediate retention host in the dissemination process. Nosocomial *bla*_NDM–1_-carrying bacteria are of concern.

Drug-resistant plasmids may be a key factor in determining drug resistance. The p11106 and p12 plasmids containing the *bla*_NDM–1_ gene isolated from *K. pneumoniae* exhibit the characteristics of the pNDM-BTR plasmid and have a polymorphic gene environment. The existence of *ble* in the surrounding environment of *bla*_NDM–1_ suggests that *bla*_NDM–1_ is derived from *A. baumannii*. Meanwhile, the presence of *TnpA* in both the upstream and downstream regions of *bla*_NDM–1_ indicates that *bla*_NDM–1_ may be acquired from gene transposition and persists over a long period in *K. pneumoniae* strains.

## Data Availability Statement

The datasets generated for this study are available from the corresponding author upon reasonable request.

## Ethics Statement

Written informed consent was obtained from all participants, and the purpose and procedures of the study were explained to them. Ethical approval for this study was obtained from the Institutional Review Board of The First Affiliated Hospital of Nanchang University.

## Author Contributions

TX and CC designed the study, collected and analyzed the data, and drafted the manuscript. TX, CC, JW, YL, and QZ contributed to the performance of the experiment and data collection. NC and XW reviewed the study design. WZ contributed to the review of data analysis and data interpretation. All authors have approved the final version of the manuscript.

## Conflict of Interest

The authors declare that the research was conducted in the absence of any commercial or financial relationships that could be construed as a potential conflict of interest.

## Footnotes

1 http://blast.ncbi.nlm.nih.gov/Blast.cgi
