# Supplementary material for: Resistance of Klebsiella pneumoniae Strains Carrying blaNDM–1 Gene and the Genetic Environment of blaNDM–1
Source: Front Microbiol. 2020 Apr 30;11:700. doi: 10.3389/fmicb.2020.00700 (PMC7203411; doi:10.3389/fmicb.2020.00700)
Supplement: Supplementary file 1 [file Table_1.DOC]

**Supplement**

**Supplement** Table 1. Patient Records

| Strains | Sources | Departments | Districts |
| --- | --- | --- | --- |
| Eco1CR | urine | Infectious Department Ward II | Nanchang, Jiangxi Province |
| Eco2CR | urine | Secretory Surgery Ward I | Shangrao, Jiangxi Province |
| Ab1CR | sputum | Neonatal Intensive Care Unit | Anqin, Anhui Province |
| Ab2CR | sputum | Respiratory Intensive Care Unit | Shangrao, Jiangxi Province |
| Ab3CR | sputum | Cadre Ward II | Nanchang, Jiangxi Province |
| Ab4CR | sputum | Neurosurgery Intensive Care Unit | Yichun, Jiangxi Province |
| Ab5CR | urine | Secretory Surgery Ward I | Fuzhou, Jiangxi Province |
| Ab6CR | blood | Blood Disease Ward II | Jiujiang, Jiangxi Province |
| Ab7CR | sputum | Respiratory Ward I | Nanchang, Jiangxi Province |
| Ab8CR | blood | Burn Intensive Care Unit | Jiujiang, Jiangxi Province |
| Kpn1CR | others | Burn Ward IV | Jingdezhen, Jiangxi Province |
| Kpn2CR | ascites | Digestion Ward IV | Jiujiang, Jiangxi Province |
| Kpn3CR | sputum | Neurology Intensive Care Unit | Nanchang, Jiangxi Province |
| Kpn4CR | blood | Burn Ward IV | Fuzhou, Jiangxi Province |
| Kpn5CR | sputum | Nephrology Ward I | Nanchang, Jiangxi Province |
| Kpn6CR | sputum | Respiratory ward II | Nanchang, Jiangxi Province |
| Kpn7CR | urine | Neurosurgery Ward I | Nanchang, Jiangxi Province |
| Kpn8CR | blood | Burn Intensive Care Unit | Jiujiang, Jiangxi Province |
| Kpn9CR | urine | Neurosurgery Ward I | Nanchang, Jiangxi Province |
| Kpn10CR | sputum | cardiovascular disease Ward III | Nanchang, Jiangxi Province |
| Kpn11CR | sputum | Blood Disease Ward II | Shangrao, Jiangxi Province |
| Kpn12CR | others | Burn Ward IV | Jingdezhen, Jiangxi Province |
| Kpn13CR | sputum | Neurosurgery Intensive Care Unit | Nanchang, Jiangxi Province |
| Kpn14CR | sputum | Department of Respiration | Nanchang, Jiangxi Province |
| Kpn15CR | urine | Peadiatrics | Fuzhou, Jiangxi Province |
| Kpn16CR | sputum | Respiration Unit | Xinyu, Jiangxi Province |
| Kpn17CR | sputum | ICU | Nanchang, Jiangxi Province |
| Kpn18CR | sputum | Respiration Unit | Pingxiang, Jiangxi Province |
| Kpn19CR | urine | Burn Unit | Jiujiang, Jiangxi Province |
| Kpn20CR | blood | ICU | Nanchang, Jiangxi Province |
| Kpn21CR | urine | Burn Unit | Shangrao, Jiangxi Province |
| Kpn22CR | sputum | Respiration Unit | Nanchang, Jiangxi Province |
| Kpn23CR | urine | Burn Unit | Nanchang, Jiangxi Province |
| Kpn24CR | urine | Burn Unit | Jian, Jiangxi Province |
| Kpn25CR | abscess | Coronary Care Unit | Pingxiang, Jiangxi Province |
| Kpn26CR | ascites | Transplant Care Unit | Jingdezhen, Jiangxi Province |
| Kpn27CR | secreta | Burn Unit | Yingtan, Jiangxi Province |
| Kpn28CR | sputum | Burn Unit | Fuzhou, Jiangxi Province |
| Kpn29CR | blood | ICU | Jiujiang, Jiangxi Province |
| Kpn30CR | sputum | Burn Unit | Jian, Jiangxi Province |
| Kpn31CR | urine | Orthopedics | Nanchang, Jiangxi Province |
| Kpn32CR | blood | Burn Unit | Yichun, Jiangxi Province |
| Kpn33CR | abscess | Emergency | Nanchang, Jiangxi Province |

**Supplement Table 2. Information and drug-resistant data of sequenced plasmids**

| Antibiotics | *Kp.* 7^a^ | *Kp.* 11 ^a^ | *Kp.*12 ^a^ | *Kp.* 24 ^a^ | *Kp.* 32 ^a^ |
| --- | --- | --- | --- | --- | --- |
|  | P7-1973^b^ | p11106 ^b^ | P12 ^b^ | P243323 ^b^ | P32 ^b^ |
| Imipenem | R | R | R | R | R |
| Meropenem | R | R | R | R | R |
| Ertapenem | R | R | R | R | R |
| Amikacin | S | I | I | S | S |
| Amoxicilin/Clavulanic acid | S | R | R | S | R |
| Aztreonam | S | R | R | R | S |
| Cefatriaxone | R | R | R | R | R |
| Ceftazidime | R | R | R | R | R |
| Cefotaxime | R | R | R | R | R |
| Cefoxitin | R | R | R | R | R |
| Cefazolin | R | R | R | R | R |
| Ciprofloxacin | R | R | R | S | R |
| Gentamicin | R | R | R | R | R |
| Levofloxacin | I | R | R | S | R |
| Piperacillin/Tazobactam | S | R | R | R | R |
| Piperacillin | R | R | R | R | R |
| Compound Xinnuoming | R | I | I | S | R |
| Tetracycline | I | R | R | R | R |
| Tekacillin/Clavulanic acid | R | R | R | R | R |
| Tobramycin | R | R | R | I | S |

^a^ Strains; ^b^ Plasmids
